# Diabetic dyslipidaemia

**DOI:** 10.1016/j.plabm.2021.e00248

**Published:** 2021-07-18

**Authors:** Subashini C. Thambiah, Leslie Charles Lai

**Affiliations:** a Department of Pathology, Faculty of Medicine and Health Sciences, Universiti Putra Malaysia, Selangor, Malaysia; b Department of Medicine, Gleneagles Kuala Lumpur, Kuala Lumpur, Malaysia

**Keywords:** Diabetic dyslipidaemia, Cardiovascular disease, Insulin resistance, Biomarkers, Lipid-lowering therapy, Guidelines

## Abstract

Diabetes mellitus (DM) is an escalating pandemic and an established cardiovascular risk factor. An important aspect of the interaction between DM and atherosclerotic cardiovascular disease (ASCVD) is diabetic dyslipidaemia, an atherogenic dyslipidaemia encompassing quantitative [hypertriglyceridaemia (hyperTG) and decreased high density lipoprotein cholesterol (HDL)] and qualitative [increased small dense low density lipoprotein cholesterol (sdLDL) particles, large very low density lipoprotein cholesterol (VLDL) subfraction (VLDL1) and dysfunctional HDL] modifications in lipoproteins. Much of the pathophysiology linking DM and dyslipidaemia has been elucidated. This paper aims to review the pathophysiology and management of diabetic dyslipidaemia with respect to ASCVD. Briefly, the influence of diabetic kidney disease on lipid profile and lipid changes causing type 2 diabetes mellitus are highlighted. Biomarkers of diabetic dyslipidaemia, including novel markers and clinical trials that have demonstrated that non-lipid and lipid lowering therapies can lower cardiovascular risk in diabetics are discussed. The stands of various international guidelines on lipid management in DM are emphasised. It is important to understand the underlying mechanisms of diabetic dyslipidaemia in order to develop new therapeutic strategies against dyslipidaemia and diabetes. The various international guidelines on lipid management can be used to tailor a holistic approach specific to each patient with diabetic dyslipidaemia.

## 1 Introduction

Diabetes mellitus (DM) is a pandemic with numbers anticipated to increase from 463 million in adults aged 20–79 years in 2019 to 700 million by 2045 [1]. Following the Framingham Study, diabetes became an established cardiovascular disease (CVD) risk factor [2]. The Organization to Assess Strategies for Ischemic Syndromes (OASIS) study demonstrated that diabetics without CVD had equivalent long-standing morbidity and mortality as non-diabetics with CVD [3]. Adults with DM compared with those without the disease are two to four times more likely to have CVD [4]. Factors associated with this cardiovascular (CV) risk include a combination of modifiable (obesity, hyperglycaemia, hypertension, dyslipidaemia) and non-modifiable (age, race, gender, genetics) variables [5]. An important aspect of this interaction includes diabetic dyslipidaemia, an atherogenic dyslipidaemia encompassing quantitative and qualitative modifications in lipoproteins, characterised by hypertriglyceridaemia (hyperTG), reduced high density lipoprotein cholesterol (HDL), elevated small dense low density lipoprotein cholesterol (sdLDL) particles, postprandial dyslipidaemia and raised remnant lipoproteins [6]. This paper aims to review the pathophysiology and management of diabetic dyslipidaemia with respect to atherosclerotic cardiovascular disease (ASCVD).

Patients with type 1 diabetes mellitus (T1DM) under good control with insulin have comparable lipid profiles to the general population. However, poorly-controlled T1DM has hyperTG, low HDL, raised low density lipoprotein cholesterol (LDL) and sdLDL particles [7]. Previous studies have revealed that a significant rise in CV risk in T1DM occurs with lipid profile derangement and increase in blood pressure after the manifestation of diabetic kidney disease (DKD) [[8], [9], [10]]. A study of 7479 T1DM subjects from the UK General Practice research database revealed that compared with the general population, CVD was higher in T1DM women and young subjects, occurring 10–15 years earlier [11]. Improved glycaemic control correlates with improved lipid profile [12] and enhanced survival in T1DM patients [13]. Evolving evidence emphasises the co-existence of metabolic syndrome (MetS) with T1DM, giving rise to the alleged ‘double diabetes’ increasing CV risk [14].

Lipid derangements in type 2 diabetes mellitus (T2DM) include hyperTG (fasting and post-prandial), reduced HDL and increased sdLDL, while total cholesterol (TC) and LDL may be normal or slightly raised [15]. This deranged lipid profile is present for years before the clinical diagnosis of T2DM [16]. A Swedish nationwide registry data analysis of T2DM patients revealed that the significant predictors for CV outcomes and death include smoking, reduced exercise, systolic blood pressure, increased HbA1c and LDL [17]. In the United Kingdom Prospective Diabetes Study (UKPDS), one in six newly diagnosed T2DM subjects was shown to have previous silent MI indicating that a significant CV risk exists for several years before the manifestation of biochemical hyperglycaemia [18]. Furthermore, individuals with impaired fasting glycaemia (IFG) and impaired glucose tolerance (IGT) have been found to have this atherogenic dyslipidaemia [19]. Unlike T1DM, lipid profile in T2DM remains deranged even with glycaemic control. Therefore, insulin resistance (IR) rather than impaired glycaemic control *per se* is linked to this lipid derangement [20]. IR is defined as reduced tissue response to insulin associated with hyperinsulinaemia. Insulin modulates metabolism of glucose and lipids via a transmembrane insulin receptor leading to insulin receptor substrate recruitment [21].

## 2 Pathophysiology of diabetic dyslipidaemia

**Fig. 1:**
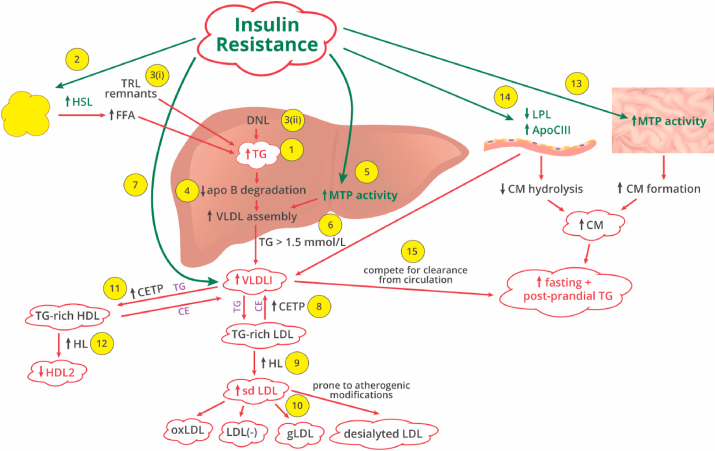
Pathogenesis of atherogenic dyslipidaemia in diabetes mellitus. 1. Hypertriglyceridaemia (hyperTG), central to the lipid abnormalities that occur in diabetic dyslipidaemia, is due to both increased production and decreased clearance [22]. 2. Insulin resistance (IR) stimulates hormone-sensitive lipase (HSL)-mediated lipolysis, which releases free fatty acids (FFA) from adipose tissue, increasing hepatic triglyceride (TG) production [24]. 3. The contribution of (i) TRL (TG-rich lipoprotein) remnants [chylomicron (CM) and very low density lipoprotein cholesterol (VLDL) remnants] and (ii) hepatic de novo lipogenesis (DNL) to hepatic TG production is minimal [23]. 4. When hepatic TG is increased, degradation of apolipoprotein (Apo) B is reduced, and VLDL production is facilitated [23]. 5. IR stimulates hepatic microsomal TG transfer protein (MTP) activity and thereby enhances VLDL assembly [23]. 6. When TG > 1.5 mmol/L, large TG-rich VLDL particles (VLDL1) form [34]. 7. In IR, VLDL1 (rich in TG, ApoCIII and ApoE) is preferentially produced compared to VLDL2 (TG-poor) [25]. 8. Cholesteryl ester transfer protein (CETP) facilitates the replacement of cholesteryl ester (CE) in low density lipoprotein cholesterol (LDL) by TG from VLDL1 resulting in TG-rich LDL [35]. 9. Lipoplysis of TG- rich LDL is increased as it is the preferred substrate for hepatic lipase (HL) resulting in small dense LDL (sdLDL) formation [35]. 10. Prolonged circulation time of sdLDL results in several atherogenic modifications of sdLDL particles [oxidised LDL (oxLDL), desialylated LDL, electronegative LDL [LDL(-)] and glycated LDL (gLDL)] in plasma [40]. 11. CETP mediates the replacement of CE in high density lipoprotein (HDL) by TG from VLDL1 to form TG-rich HDL [15]. 12. Being a thermodynamically unstable particle, the catabolism of TG-rich HDL is accelerated by HL resulting in low HDL2 [15]. 13. IR stimulates intestinal MTP activity and thereby increases CM formation [6]. 14. IR diminishes lipoprotein lipase (LPL) activity located in the luminal surface of the capillary endothelium (skeletal and cardiac muscle, adipose tissue and lactating breast) and increases ApoCIII secretion resulting in decreased hydrolysis of CM and VLDL [6]. 15. CM (ApoB48) and VLDL (ApoB100) particles compete for clearance as both are cleared from the circulation by common pathways, further aggravating fasting and postprandial hyperTG [15].

Lipoprotein abnormalities in DM include quantitative (hyperTG and decreased HDL) and qualitative changes [increased sdLDL particles, large very low density lipoprotein cholesterol (VLDL) subfraction (VLDL1) and dysfunctional HDL]. HyperTG, central to the lipid abnormalities that occur in diabetic dyslipidaemia, is due to both increased production and diminished clearance [22]. Three distinct pathogenesis secondary to abnormal glucose metabolism have been postulated that provide important fundamental knowledge for understanding diabetic hyperTG. In IGT, the loss of insulin sensitivity leads to compensatory increase in insulin secretion, and hence, increased hepatic VLDL production. In T2DM with relative insulin deficiency, raised free fatty acids (FFA) stimulate hepatic VLDL production. However, in T1DM, the hyperTG is mainly due to defective VLDL removal because the liver cannot respond to raised FFA under absolute insulin deficiency. Hence, hepatic VLDL production is not stimulated by raised FFA [23]. The pathogenesis of diabetic dyslipidaemia is illustrated in Fig. 1.

### 2.1 HyperTG

Circulating FFA play a vital role in hepatic TG production and are derived mainly from adipose tissue. The contribution of chylomicrons (CM) and VLDL remnants and hepatic *de novo* lipogenesis (DNL) to TG production is minimal [23]. IR stimulates hormone sensitive lipase (HSL)-mediated lipolysis, which releases FFA from adipose tissue TG, increasing hepatic TG production [[24], [25], [26]]. IR also stimulates VLDL production by enhancing hepatic microsomal TG transfer protein (MTP) activity that transfers neutral lipids to nascent apolipoprotein (Apo) B. VLDL particles differ in composition and size and is categorised by their diameter, density, and flotation [16]. In IR, VLDL1 (rich in TG, ApoCIII and ApoE) is preferentially produced compared to VLDL2 (TG-poor) promoting increased remnant particles, sdLDL, reduced HDL, and compositional changes in HDL [25,26]. The concept of VLDL1 and VLDL2 is derived from mathematical multicompartmental modelling studies of lipoprotein metabolism but this has not been validated by particle composition studies [16]. The combination of hyperglycaemia, IR, and hyperinsulinaemia appear to stimulate lipogenesis through simultaneous upregulation of transcription factors, carbohydrate responsive element binding protein (ChREBP) and sterol response element binding protein-1c (SREBP-1c). Insulin stimulates hepatic DNL through SREBP-1c whereas hyperglycaemia stimulates VLDL enlargement via ChREBP [25,26]. Reduced adiponectin in IR may have a role in the pathogenesis of diabetic dyslipidaemia by increasing FFA availability for VLDL production [25].

HyperTG is further augmented by decreased VLDL catabolism secondary to reduced lipoprotein lipase (LPL) activity and increased serum ApoCIII due to IR [27]. IR stimulates hepatic ApoCIII expression by Forkhead box protein O1 activation [23]. LPL facilitates TG hydrolysis in CM or VLDL releasing monoacylglycerol and FFA. LPL also promotes the uptake of FFA by adipocytes [21]. Other regulators of LPL include ApoAV and angiopoietin-like proteins (ANGPTLs) but the role of IR in affecting these molecules to increase TG-rich lipoprotein (TRL) catabolism remains to be elucidated [23]. Imbalance in the processes involved in hepatic lipid homeostasis leads to non-alcoholic fatty liver disease (NAFLD) due to increased VLDL secretion or lipid accumulation in hepatocytes [28].

The postprandial TG peak in T2DM or IR is due to raised intestine-derived and hepatic TRL that can be attributed to increased secretion and/or reduced clearance of lipoproteins. Dietary TG enters the endoplasmic reticulum and fuses with ApoB48 particles to form prechylomicrons mediated by increased intestinal MTP activity that is stimulated by IR. Mature CM with a single ApoB48, ApoAI and ApoAIV are then exocytosed at the basolateral membrane. Raised plasma ApoAIV is present in T2DM patients with hyperTG. The mechanism of intestinal ApoAIV in influencing postprandial lipid metabolism in T2DM remains unclear and is subject to further research [6]. ApoB48 on CM particles is 48% of the complete ApoB and does not have the ApoB portion that interacts with the LDL receptor (LDLR) [20]. Its production rate is increased in T2DM [25,29]. ApoB48 is also found in atherosclerotic lesions, indicating that CM remnants are able to form plaques by entering the endothelium [30]. Other contributing factors to increased CM production in IR include impaired glucagon-like peptide 1 (GLP-1) and increased glucagon-like peptide 2 (GLP-2) secretion, as well as leptin resistance [6].

Decreased clearance of lipoproteins in IR can be attributed to reduction in LPL activity, change in lipoprotein composition and increased competition for clearance caused by decreased removal of hepatic remnant or increased TRL pool size [31]. ApoB100- and ApoB48-comprising lipoproteins, compete for clearance from circulation by LPL hydrolysis and subsequently for hepatic remnant removal. Thus, this dysregulation of the VLDL–IDL–LDL (endogenous) and CM-CM remnant (exogenous) delipidation cascades contributes to the prolonged postprandial dyslipidaemia. Fasting and post-prandial hyperTG are further aggravated by consecutive meals where TG does not revert to baseline level before ingestion of the subsequent meal [15].

### 2.2 sdLDL

Heterogeneity of LDL particles is with regard to size, chemical composition, density and metabolism. These differences give rise to Pattern A [large buoyant LDL (lbLDL)] and Pattern B (sdLDL) [32]. Hepatic lbLDL is produced from small VLDL2 (cholesterol-rich, TG-poor) and intermediate density lipoprotein cholesterol (IDL) particles whereas sdLDL is generated from large VLDL1 (TRL) particles [33].

In IR, the LDL concentration is typically normal or only slightly raised but it is of abnormal composition (sdLDL), attributed to hyperTG. When TG > 1.5 mmol/L, VLDL1 forms and when hydrolysed by LPL, LDL particles with altered ApoB conformation are produced that remain in the circulation for a prolonged time as they fail to bind efficiently to LDLR [26,34]. The exchange of cholesteryl ester (CE) for TG in LDL mediated by cholesteryl ester transfer protein (CETP) gives rise to TG-rich LDL, which is the preferential substrate for sdLDL production by hepatic lipase (HL) [35].

The formation of distinct LDL subfractions is not fully understood. Two pathways reliant on hepatic TG availability which produce different sizes of VLDL particles have been suggested. There is metabolic channelling within the VLDL-IDL-LDL delipidation cascade which produces diverse IDL and LDL products from various TRL precursors. In the state of low TG availability, VLDL1 (TRL) and IDL2 (TG-poor lipoprotein) are secreted. Larger particles are secreted that include VLDL1 (TRL) and VLDL2 (TG-poor lipoprotein) if TG availability is high. TG-poor lipoprotein is converted into lbLDL subclasses (LDL1 and LDL2), whereas TRL is a precursor for sdLDL subclasses (LDL3 to LDL7) after delipidation by LPL and HL [36]. ApoE and ApoCIII are counter-regulators of ApoB lipoprotein metabolism which help to clarify the metabolic origin of the LDL particles. ApoE facilitates the clearance of ApoB lipoproteins from the circulation while ApoCIII blocks their clearance. ApoE-containing TG-rich VLDL is mainly secreted by the liver when plasma TG is normal and is promptly cleared from the circulation. In hyperTG, however, removal of ApoE-containing lipoprotein is reduced and ApoCIII-containing TRL are predominantly secreted that have longer circulation time and are transformed into sdLDL. This explains the significance of controlling hyperTG for reduction of CV risk [37].

The relationship between the composition and biological properties of circulating sdLDL and the risk of atherosclerosis and CVD development has been established [38]. However, the Atherosclerosis Risk in Communities (ARIC) Study reported that sdLDL particles do not remain independent predictors of CV risk when adjusted for other lipid risk factors such as LDL, ApoB, and TC. Interestingly, sdLDL demonstrated predictive power for coronary artery disease (CAD) risk in individuals with ideal LDL concentration [39]. The atherogenicity of sdLDL is explained by its reduced affinity for LDLR, hence its longer half-life, as well as its lipid and protein components that are more prone to chemical modifications and uptake by scavenger receptors [40]. It is, therefore, predicted that individuals with elevated sdLDL have approximately three to seven-fold increase in CAD risk [41]. A previous study has shown a significant association of increased CAD risk with reduced LDL peak particle size where the lowest LDL size was associated with CAD evolution on angiography [33]. sdLDL is prone to several atherogenic modifications due to its prolonged circulation time in plasma [38].

### 2.3 Atherogenic modified LDL

The primary source of cholesterol in atherosclerotic plaques is the LDL particle. Based on previous studies, lipid accumulation in cultured cells is not caused by the native LDL, but by highly atherogenic modified particles, including oxidised LDL (oxLDL), desialylated LDL, glycated LDL (gLDL) and electronegative LDL [LDL (−)]. These four forms of atherogenic LDL modifications are also prone to aggregation and complex formation that will cause increased atherogenicity and pro-inflammatory effects [38].

Oxidation in plasma produces oxidation-specific epitopes on LDL, generating an immune response and inflammation. oxLDL, but not the non-oxidised particle, accumulates in macrophages. oxLDL is taken up by arterial cells as it is unable to bind to the LDLR. A few suggested possible mechanisms for LDL oxidation of lipid constituents preferentially involve the reactive oxygen species (ROS), lipoxygenase, and metals. Myeloperoxidase, which is an oxidase generated by macrophages and neutrophils, produces both hypochlorous acid and hypothiocyanous acid which are potent oxidants that can oxidise ApoB100. Through a few mechanisms, oxLDL can trigger inflammation as it increases fat deposits, ROS generation, pro-inflammatory conditions, and apoptosis of macrophages and monocytes. Moreover, in endothelial cells, oxLDL can also initiate expression of lectin-like oxidised LDLR-1 in atherosclerotic plaques, stimulate interleukin-8 production and activate its secretion in aortic smooth muscle cells through activation of ROS-mediated signalling. Higher risk of carotid atherosclerosis can be determined by reduced levels of anti-oxidised LDL antibodies and higher oxLDL content in an individual [42].

Desialylation is one of the atherogenic modifications that occurs in blood plasma by *trans*-sialidase, which is crucial in glycoconjugate metabolism. *Trans*-sialidase transfers the part of sialic acid, a vital constituent of native LDL to plasma proteins from the LDL particle. In subjects with Pattern B, the sialic acid content of sdLDL is less than that of lbLDL. The affinity of sdLDL particles to proteoglycans is elevated by desialylation. This prolongs their subendothelial resident time leading to the development of atherosclerotic plaque [38]. Desialylated subfraction of LDL impairs CETP-mediated reverse cholesterol transport (RCT) and inhibits the esterifying activity of lecithin cholesterol acyltransferase (LCAT) in macrophages. It is also greatly immunogenic and generates pro-atherogenic immunoglobulin (Ig) G self-antibodies. These factors further contribute to atherogenesis. In atherosclerosis, desialylated LDL is significantly increased compared with healthy individuals, contributing 60% of LDL in patients with CAD. Apart from that, a marked reduction in sialylation rate correlates with elevated atherogenicity of sdLDL [43].

LDL may be irreversibly and intensively modified through non-enzymatic glycation and glyco-oxidation mechanisms in hyperglycaemia. Targets for glycation include the lipid and protein (ApoB100) moieties in LDL particles. ApoB in sdLDL is shown to be preferentially glycated (*in vitro* and *in vivo*) compared with lbLDL and a higher percentage is glycated in T2DM (14.8%) compared with non-diabetics (4.8%). In T2DM and MetS, sdLDL is prone to glycation. The LDL subfractions in T2DM are composed of pro-atherogenic gLDL and desialylated LDL. These circulating atherogenic modified LDL can induce lipid uptake via cultivated aortic smooth muscle cells and stimulate expression of receptors for advanced glycation end products (RAGE). gLDL and other advanced glycation end products (AGE) also enhance expression of other scavenger receptors in macrophages. The AGE-RAGE signalling initiation attracts monocytes to the vascular intima, induces endothelial dysfunction, increases oxidative stress, promotes vascular wall remodelling, and stimulates nuclear factor kappa light chain enhancer of activated B cell-dependent expression of pro-inflammatory and prothrombotic molecules leading to vascular damage and progression of atherosclerotic lesions [42].

A few mechanisms have been suggested for the increased plasma electronegative LDL [LDL (−)] levels in atherosclerotic subjects, including protein component modification, oxidation and binding to proteoglycans. It has been shown that increase in oxLDL and sdLDL levels are closely related to the LDL (−) production [38]. LDL (−) is rich in desialylated LDL. This suggests that desialylated and LDL (−) subfractions may be similar as both of their particles contain less antioxidants than native LDL and are susceptible to oxidation. Hence, it is reasonable that LDL has undergone numerous modifications in the bloodstream that begins with acquirement of the negative charge and desialylation, then subsequently oxidation and formation of pro-inflammatory and extremely atherogenic complexes. Up to 88% of the electronegative LDL subfractions are desialylated LDL, which is highly pro-atherogenic [44].

### 2.4 HDL

Low HDL is an independent predictor of CVD. The anti-atherogenicity of HDL is due to its role in RCT where it facilitates the transport of excess cholesterol for hepatic bile excretion from peripheral cells [15]. In addition, HDL also inhibits oxidation, reduces inflammation, affects innate immunity and improves endothelial function [45]. Uptake of free cholesterol by freshly secreted HDL is esterified by LCAT forming HDL containing CE. When TRL is present, CETP mediates the replacement of CE in HDL by TG to form TG-rich HDL, a thermodynamically unstable particle whose catabolism is accelerated by HL [15]. In T2DM, HDL is reduced whereas normal or increased HDL levels are observed in T1DM [4]. HDL can be classified based on density where the large buoyant HDL2 (cholesterol-rich) and small dense HDL3 (cholesterol-poor) are inversely and directly associated with CVD, respectively. Similar decrease in HDL2 was observed in subjects with T2DM. HDL3 in T2DM has decreased anti-oxidant activity due to hyperglycaemia, hyperTG and oxidative stress [46]. In both T1DM and T2DM, HDL RCT function is impaired [4].

In addition, HDL particles in DM can also undergo significant modifications in composition and structure, resulting in dysfunctional HDL due to systemic and vascular inflammation [45,46]. Chronic inflammation increases serum amyloid A (SAA) in T2DM and this displaces ApoAI and other proteins from the HDL surface, leading to increased clearance of HDL. This explains the low HDL levels in T2DM [45]. Carbamylated HDL is also dysfunctional and is independently associated with mortality outcomes in T2DM patients [47].

### 2.5 Pathogenesis of diabetic dyslipidaemia in T1DM

The pathogenesis described above is more related to IR in T2DM, although some mechanisms leading to quantitative and qualitative lipoprotein abnormalities are similar in both T1DM and T2DM. Quantitative lipoprotein abnormalities in T1DM are associated with glycaemic control as evident in the Diabetes Control and Complications Trial (DCCT) study. The qualitative lipoprotein abnormalities are similar to T2DM and are present in spite of optimal glycaemic control [48].

In diabetic ketoacidosis (DKA), reduced catabolism of TG-rich lipoproteins (CM and VLDL) is due to decreased LPL activity secondary to absolute insulin deficiency, giving rise to hyperTG and reduced LDL. HDL is also significantly decreased as a consequence of hyperTG, pathogenesis as in T2DM. However, well-titrated insulin treatment can reverse these quantitative lipoprotein abnormalities in DKA rapidly [48].

In patients with suboptimal or poor glycaemic control, increased TG and LDL are observed with additional reduced HDL (decrease in HDL2) in patients with albuminuria. HyperTG is due to relative insulin deficiency stimulating increased circulating FFA leading to increased VLDL production. Increased VLDL production leads to increased LDL production as TG-rich lipoprotein catabolism is not adequately decreased [48].

In optimal glycaemic control, TG and LDL are within target values or marginally reduced whereas HDL is also within target values or marginally raised. The marginal decrease in TG is due to intense insulin treatment. As a consequence of subcutaneous insulin, markedly raised plasma insulin levels downregulate VLDL production. Increased LPL activity due to peripheral hyperinsulinaemia also contributes to low TG. Similarly, the low LDL is a direct consequence of decreased VLDL production. Marginally increased HDL was reported to be due to raised LPL activity with normal HL activity (raised LPL:HL) due to peripheral hyperinsulinaemia [48].

## 3 Influence of diabetic kidney disease (DKD) on lipid profile

Lipid profile changes significantly as DKD progresses [26]. Albuminuria increases plasma FFA levels that stimulate hepatic TG synthesis and VLDL production. The increased levels of TRL enhance CE transfer and decrease HDL. DKD is an independent causative factor for dyslipidaemia and it exacerbates lipid disorders in diabetics [21]. The incidence of CVD considerably increases with the progression of DKD. Plasma von Willebrand factor (VWF), a surrogate marker for vascular endothelial damage, is significantly elevated in albuminuric diabetes and not in nondiabetic patients with kidney disease, suggesting that albuminuria in diabetes indicates extensive vascular endothelial damage in addition to kidney damage [23]. In nephrotic syndrome, angiopoietin-like 4 (ANGPTL4) is thought to play a role in hyperTG development [49]. Its role in dyslipidaemia associated with DKD, however, remains to be elucidated [23].

## 4 Lipid changes causing T2DM

Lipid derangement may also cause T2DM. The exact pathogenesis is only partly understood but it involves mainly hyperTG and reduced HDL. HyperTG gives rise to increased FFA levels that lead to disruption of the cascade connecting insulin receptors with glucose transporters and subclinical inflammation. This induces IR and beta (β)-cell dysfunction. This explains the challenge in regulating hyperglycaemia in patients with hyperTG compared with normal TG levels. It also clarifies the decreased intensity in anti-diabetic treatment once hyperTG has resolved [50].

The role of HDL in RCT mediating cholesterol efflux from various tissues is thought to alter the intracellular lipid environment. Hence, low HDL may cause micro-inflammation leading to reduced insulin sensitivity and secretion [50].

## 5 Biomarkers of diabetic dyslipidaemia

Lipid investigations are performed to assess CV risk and monitor treatment [51]. The seemingly ‘normal’ serum cholesterol level in both T1DM and T2DM masks an atherogenic lipid profile with sdLDL and dysfunctional, low HDL level [52].

Conventionally, plasma lipid analyses have required fasting samples. Studies have demonstrated that TG level is approximately 0.3 mmol/L higher in a non-fasting sample, which is of no clinical significance in most individuals [53]. Fasting is also not required for ApoB measurement as the concentration of ApoB48-comprising CM represents <1% of the total ApoB-comprising lipoproteins circulating in the post-prandial state [54]. However, if non-fasting sampling is used, calculated LDL must be interpreted cautiously in individuals with DM, MetS, or hyperTG [51].

### 5.1 Conventional lipid profile

Lipoprotein concentrations are generally not directly measured in clinical practice but are estimated from their cholesterol content. A conventional serum lipid profile report consists of concentrations of TC, LDL, HDL, and TG where LDL is usually calculated using the Friedewald formula. This formula has its limitations as it cannot be used when TG > 4.5 mmol/L. It is prone to methodological errors as it includes measurements of TC, HDL, TG and assumes that the TC:TG ratio is constant in VLDL [51]. Calculated LDL also underestimates LDL concentration when TG ≥ 2.0 mmol/L, and is misleading at extremely low LDL concentrations with hyperTG [55]. To overcome these issues, direct measurement of LDL by enzymatic methods has been established. Nevertheless, limitations of direct LDL measurement include inaccuracy and systematic bias in dyslipidaemic subjects, particularly for hyperTG [56].

For more accurate LDL estimation to be used routinely, the Martin/Hopkins equation was established with the same lipid parameters (TC, TG, HDL) as the Friedewald equation, but instead of a fixed conversion factor, it uses a patient-specific factor for LDL calculation. This Martin/Hopkins equation was developed using density gradient ultracentrifugation in subjects with a wide range of LDL values and included those with and without lipid-lowering treatment. The accuracy of the Friedewald and Martin/Hopkins LDL equations were compared with preparative ultracentrifugation (gold standard) in subjects with Friedewald LDL < 1.03 mmol/L (40 mg/dL) in the Further Cardiovascular Outcomes Research With PCSK9 Inhibition in Subjects With Elevated Risk (FOURIER) trial. The results demonstrated that the Martin/Hopkins equation more closely approximates preparative ultracentrifugation than the Friedewald equation in patients achieving low LDL with proprotein convertase subtilisin/kexin 9 (PCSK9) inhibitors. This indicates that the Martin/Hopkins equation may prevent under-treatment compared with Friedewald equation that underestimates LDL [57].

Another LDL equation was developed more recently using β-quantification results from hyperTG patients that can accurately estimate LDL in patients with TG up to 9.03 mmol/L (800 mg/dL). It also appears to be more accurate than other equations for normolipidaemic patients. Moreover, compared to other equations, it correctly categorises dyslipidaemic patients into different LDL treatment groups, improving the use of calculated LDL in management of CVD risk [58].

### 5.2 Non-HDL and ApoB

As an alternative, non-HDL (TC minus HDL), is a simple measure of cholesterol in all atherogenic lipoproteins. Being a calculated value, there is no additional cost and fasting samples are not required [59]. Non-HDL should be reported in all standard lipid panels [54].

An ideal measure of atherogenicity would be direct quantitation of ApoB given its central role in the pathogenesis of atherosclerosis. Irrespective of density or cholesterol content, all ApoB-comprising lipoproteins have a single ApoB molecule. Hence, ApoB quantitation directly evaluates the number of atherogenic particles in plasma. Measuring ApoB concentration will provide a more discerning index of estimating CV risk and guiding therapeutic decision than LDL [60]. In addition, the methods for ApoB have better analytical performance compared with measured or calculated LDL and non-HDL [54].

Non-HDL and ApoB are advocated for CV risk assessment, especially in individuals with hyperTG, DM, MetS, obesity, or extremely low LDL concentrations [51]. Since hyperTG is common in DM, both these lipid parameters can be used to guide therapy. The specific therapeutic goal for non-HDL should be 0.8 mmol/L above the corresponding LDL goal. An ApoB of <0.8 g/L is considered optimal in diabetics [61].

However, there is controversy about which is better; non-HDL or ApoB. The Emerging Risk Factors Collaboration (ERFC) trial revealed that non-HDL predicts CV risk better than LDL [62]. A meta-analysis of statin-induced variations in LDL, ApoB and non-HDL showed that during statin treatment, ApoB was consistently associated with outcomes compared with non-HDL or LDL [63]. Adding ApoB to LDL, however, in a subgroup of 37 studies of 165,544 participants, added little to risk prediction [64].

### 5.3 ApoA1 and ApoB/ApoA1

ApoAI is the surrogate marker for circulating HDL particles, where there are two to four molecules per HDL particle [65]. The ApoB/ApoAI ratio has been proposed to be a strong CV risk predictor [66,67]. However, ApoAI measurement is not as widely available and standardised compared with ApoB. These issues limit the feasibility of using both ApoA1 and ApoB/ApoAI ratio in clinical decision making [65].

### 5.4 LDL subfractions

Conventional lipid profile measures total LDL (sum of cholesterol in all LDL subfractions). A substantial percentage of individuals who have had a CV event were reported to have normal LDL levels [32]. Individuals with sdLDL (Pattern B) will be at greater CAD risk compared to subjects with lbLDL (Pattern A) and their LDL is usually within target value or borderline normal. Normal LDL concentrations with Pattern B in patients with CAD [32,68] represent a hidden atherogenic risk when measuring conventional lipid profile instead of LDL subfractions.

Measurement of LDL subfractions may offer added predictive power to estimate CV risk when combined with LDL alone or other risk factors [69]. However, no prospective studies have particularly assessed the benefits on CVD if particle size patterns are changed [4]. The Veterans Affairs High-Density Lipoprotein Cholesterol Intervention Trial (VA-HIT) analysis, however, does propose a role for this mechanism [70].

No reference method exists for LDL subfractions presently. Current methods such as nuclear magnetic resonance, density gradient ultracentrifugation, and non-denaturing polyacrylamide gradient gel electrophoresis (PGGE) are expensive, laborious, time-consuming and not user-friendly [71]. In addition, these methods are not standardised. Hence, result comparability cannot be extrapolated [72]. A systematic review showed a broad range of variability of concordance in the categorisation of LDL particle patterns from 7 to 94% [69]. However, the Food and Drug Administration (FDA) has approved an alternative, less challenging method for lipoprotein subfractions which uses a modified PGGE technique termed polyacrylamide gel electrophoresis (PAGE). This standardised, operator-friendly, less expensive and quick method separates LDL particles on the basis of size into seven subfractions (lbLDL1 and 2, and sdLDL3 to 7), making it a more favourable method for the routine laboratory than PGGE [72].

### 5.5 Lp(a)

Lp(a) is a pro-thrombotic factor due to having a structure that is similar to plasminogen that leads to increased thrombosis when it binds to the plasminogen receptor [51]. There are conflicting studies on the association of Lp(a) with T2DM. Some show a direct association between Lp(a) and the incidence of T2DM [73], while others reveal an inverse relationship [74] and no association [75]. This association is further complicated by the fact that a weak correlation exists between Lp(a) and other lipid derangements [76]. Lp(a) measurement should be done at least once in a lifetime to recognise a hereditary, very high Lp(a) > 430 nmol/L that is related to a considerably high CV risk [51].

### 5.6 Novel lipid biomarkers

New biomarkers of CV risk such as modified plasma LDL [oxLDL, AGE-LDL and LDL (−)] are being explored to aid in estimating the extent and progression of atherosclerotic lesions. For this purpose, several assays have been developed to evaluate these biomarkers. However, conclusive studies are lacking as there is currently no assay standardisation. A significant progress in using modified LDL as a biomarker is the quantitation of immune complexes (IC) formed by antibodies and modified LDL (oxLDL-IC or AGE-LDL-IC) in T1DM patients. Detection of specific autoantibodies against oxLDL, LDL (−) and AGE-LDL in plasma has been associated with the presence of ASCVD. The current evidence suggests that IgG antibodies are directly linked with atherosclerosis, whereas IgM antibodies are protective. However, the technical complexity of the IC precipitation step prior to its quantitation by immunoassay renders it cumbersome to implement in most diagnostic laboratories [77].

## 6 Management

Management of diabetic dyslipidaemia includes non-pharmacological and pharmacological treatment. Non-pharmacological therapy involves dietary change and exercise. Improvement in lipid profile (decrease in TG, increase in HDL), glycaemic control and IR is associated with a 5% decrease in body weight [22]. Two prospective, controlled, long-term studies on the association between weight loss and CV outcomes were Actions for HEAlth in Diabetes (Look AHEAD) [78] and Swedish Obese Subjects (SOS) studies [79]. The Look AHEAD study revealed that, among overweight and obese T2DM patients, intervention with an intensive lifestyle focusing on weight loss did not decrease CV event rate when compared with a diabetes support and education control programme [78]. In contrast, the SOS study demonstrated that bariatric surgery in obese adults was associated with CV death and CV event reduction, respectively, compared with usual care [79].

### 6.1 Glycaemic control

The risk of CVD in non-diabetics is significantly reduced when fasting plasma glucose is 4.0–4.9 mmol/L [4]. However, both the DCCT [80] and the UKPDS [81] did not show significant beneficial macrovascular outcomes with more intensive glycaemic control. Furthermore, both the Action in Diabetes and Vascular Disease: Preterax and Diamicron Modified Release Controlled Evaluation (ADVANCE) [82] and Action to Control Cardiovascular Risk in Diabetes (ACCORD) [83] studies failed to demonstrate a positive outcome on CVD in T2DM when near-normal glucose concentrations were targeted using HbA1c < 6.5%. More disturbing was the ACCORD study finding that near-normal glycaemic control was significantly associated with an increased risk of death [84].

Nevertheless, the Epidemiology of Diabetes Interventions and Complications (EDIC) follow-up study from the DCCT showed that the original tight glycaemic control was related to long-standing benefits in CAD reduction [85] that persisted for up to 30 years [10]. Similarly, a 10-year follow up study from the UKPDS showed a ‘legacy’ effect of the period of intensive glucose lowering. Participants from the intensive group had an ongoing significant risk reduction for microvascular complications as well as macrovascular outcomes, which were previously non-significant [86].

A recent network meta-analysis on comparative effectiveness of glucose-lowering drugs on CV risk in T2DM revealed that no treatment differs from placebo for vascular outcomes in T2DM patients at low CV risk. However, specific GLP-1 receptor agonists and sodium–glucose cotransporter 2 (SGLT2) inhibitors have a favourable effect on some CV outcomes in T2DM patients at increased CV risk on metformin-based background therapy [87].

### 6.2 Effects of glucose-lowering and other therapies on lipoproteins

Studies have demonstrated that non-lipid lowering therapies can lower CV risk in diabetics [88]. Insulin therapy in T1DM can reduce TG and TRL, especially in patients with poor glycaemic control [89], whereas sulfonylureas, peroxisome proliferator-activated receptor gamma (PPAR) agonist (thiazolidinediones), SGLT2 inhibitors, dipeptidyl-peptidase 4 (DPP4) inhibitors, GLP-1 receptor agonists and metformin can significantly reduce TG [4]. Metformin may diminish CM secretion indirectly by increasing GLP-1 production or delaying gastric emptying. In T2DM, timing of metformin delivery can change post-prandial TG where the pre-meal dose decreases it more than the post-meal dose [6].

Pioglitazone, a PPARγ agonist, is a potent insulin sensitiser and is believed to reduce hepatic ApoCIII production resulting in increased LPL activity and more rapid VLDL clearance [90]. In T2DM subjects, pioglitazone demonstrated a significant decrease in CV events in the PROspective pioglitAzone Clinical Trial In macroVascular Events (PROACTIVE) study [91] unlike rosiglitazone in the Rosiglitazone Evaluated for Cardiac Outcomes and Regulation of Glycemia in Diabetes (RECORD) trial, did not increase overall CV events compared with standard glucose-lowering therapy [92]. Both increase the risk of heart failure with rosiglitazone increasing the risk of fractures, particularly in women [91,92].

SGLT2 inhibitors lower TG but also both LDL and HDL levels slightly [52]. Recent trials have demonstrated improved CV outcomes in T2DM on canaglifozin, dapaglifozin and empaglifozin. Potential indirect CV protective benefits of SGLT2 inhibitors on CM secretion have been suggested [6]. The Empagliflozin Cardiovascular Outcome Event Trial in T2DM Patients-Removing Excess Glucose (EMPA-REG) outcome trial of empagliflozin in T2DM subjects with established CVD showed a significant decrease in CV events and overall mortality. The slight increase in LDL suggested that decrease in CV events with empagliflozin is not associated with dyslipidaemic effects [93].

GLP-1 receptor agonists improve CVD outcomes in T2DM, in part by its effect on intestinal CM secretion. Studies on lixisenatide and liraglutide treatments in T2DM revealed that the effect of GLP-1 agonists is on both the production and clearance of ApoB48, hence lowering postprandial TG [6]. Increased dosage has been sanctioned for weight decrease [4]. Anagliptin, a DPP4 inhibitor, may inhibit hepatic cholesterol synthesis. A phase III trial revealed that LDL decreased by 0.25 mmol/L over a 12-week period, irrespective of statin use [94].

In the XENical in the Prevention of Diabetes in Obese Subjects (XENDOS) study involving obese subjects, orlistat, a gastrointestinal lipase inhibitor that prevents the absorption of dietary fats, significantly reduced the risk of T2DM as a result of weight reduction [95]. For hypertension treatment in diabetic dyslipidaemia, some drugs are preferable such as angiotensin II receptor blockers, angiotensin-converting enzyme inhibitors and calcium-channel blockers (lipid neutral) and alpha (α)-blockers (lipid friendly) compared with β-blockers or thiazide diuretics (lipid hostile) [96].

### 6.3 Lipid-lowering therapy

Considerable evidence demonstrates that lipid lowering therapy significantly decreases CAD in all individuals, regardless of whether they have DM. LDL is recognised as the primary target of therapy. There is also no lowest threshold whereby a further decline in LDL might be beneficial [97,98]. In contrast to hyperglycaemia, targeting dyslipidaemia appears to significantly prevent macrovascular complications in people with DM. Individuals with IGT are at significant risk of CVD and therefore, should be treated with lipid-lowering therapy although no clinical trials have been exclusively carried out in this population [65].

#### 6.3.1 Statins

Statins are the first line treatment for dyslipidaemia, unless contraindicated [99]. Statins inhibit the rate-limiting enzyme in cholesterol biosynthesis, which is 3-hydroxy-3-methylglutaryl-coenzyme A reductase (HMG-CoA reductase). Reduced hepatic cholesterol levels upregulate LDLR expression leading to decreased plasma LDL [100]. TRL contain many ApoE molecules and one ApoB. Statins can decrease TG by acting on LDLR as it acts as a receptor for ApoB at high affinity and ApoE at low affinity [4]. Other pleiotropic effects of statins include reduced oxidative stress and vascular inflammation with enhanced endothelial function [22].

Clinical trials have revealed the benefits of statins in diabetic patients in lowering LDL, regardless of its baseline value and in both the primary and secondary prevention of CVD. The Cholesterol Treatment Trialists’ (CTT) prospective meta-analysis of data on 90,056 participants in 14 randomised control trials (CT) of statins revealed that the 5-year incidence of coronary revascularisation, major coronary events, and stroke can be reduced by one fifth per mmol/L reduction in LDL with statin treatment, regardless of the initial lipid profile or other presenting features [97]. The CTT meta-analysis highlighted that, regardless of baseline LDL, there was an approximately 20% decrease in CV events for every 1.0 mmol/L decrease in LDL with statin treatment. Hence, statin therapy should be commenced in diabetics who have high CV risk, unless contraindicated [98].

The risk reduction by statins is dose-dependent, with increased doses correlating with a higher reduction of CV events [52]. All guidelines recommend statins as first-line therapy for diabetic dyslipidaemia [51,65,99,[101], [102], [103], [104]]. The intensity of statin treatment is categorised as follows: high, moderate and low with regard to LDL percentage reduction (Table 1) [102]. Statins are mostly well-tolerated although myalgia, the most frequent side-effect, may affect 5%–10% patients [22].

**Table 1:** Intensity of statin treatment with regard to LDL-reduction [102].

Intensity	High	Moderate	Low
LDL-reduction	≥50%	30%–49%	<30%
Statin	Atorvastatin 40–80 mg Rosuvastatin 20–40 mg	Atorvastatin 10–20 mg Fluvastatin 40 mg bd Fluvastatin XL 80 mg Lovastatin 40–80 mg Pitavastatin 1–4 mg Pravastatin 40–80 mg Rosuvastatin 5–10 mg Simvastatin 20–40 mg	Simvastatin 10 mg Lovastatin 20 mg Fluvastatin 20–40 mg Pravastatin 10–20 mg

##### 6.3.1.1 Statins and new onset diabetes

Chronic statin use was first shown to be linked with an increased risk of developing DM in the Justification for the Use of Statins in Primary Prevention: An Intervention Trial Evaluating Rosuvastatin (JUPITER) study. This trial involved 17,802 women and men where DM in the group which received rosuvastatin 20 mg/day was increased by 0.6% [105]. This increased risk of new onset DM was verified in a Finish observational study assessing 8749 non-diabetic men [106] and the Women's Health Initiative study [107]. One meta-analysis demonstrated that the lower the LDL level reached, the higher the odds ratio (OR) of developing T2DM (OR was 1.33, 1.16, and 1.01 for LDL levels achieved < 1.8 mmol/L, 1.8–2.59 mmol/L and >2.59 mmol/L, respectively) [108]. In contrast, the West of Scotland Coronary Prevention Study (WOSCOPS) demonstrated a reduction in the incidence of new-onset DM with pravastatin treatment [109].

Data from 20 randomised CTs confirmed that statin therapy increased the risk of incident T2DM (OR 1.12, 95% CI 1.06–1.18) than in controls. In the same study, using mendelian randomisation, the researchers showed that reduced HMG-CoA-reductase activity marginally increased T2DM risk (rs17238484-G allele OR per allele 1.02, 95% CI 1.00–1.05; rs12916 allele 1.06, 95% CI 1.03–1.09), thus inferring that statins partly confer an increased T2DM risk. It was also revealed that reduced LDL and increased T2DM risk alleles were associated with raised BMI (increase of 0.11 kg/m^2^ with rs17238484-G allele versus controls, 95% CI 0.07–0.14, p = 1.77 × 10^–7^). Weight gain caused by statins (increase of 0.30 kg, 95% CI 0.18–0.43, p = 3.15 × 10^–6^) was also noted. Weight gain is physiologically associated with IR and is a significant T2DM risk factor, and may explain the higher risk of T2DM in patients on statin treatment [110].

Although all statins may induce T2DM onset, this risk occurs in a dose dependent manner and appears higher with atorvastatin and rosuvastatin compared with pravastatin and lovastatin, reflecting the influence of differences in potency, organ specificity, and lipophilic/hydrophilic properties [111]. Despite this increased incidence, guidelines stress that the CV benefits of statins far offset the risk for developing T2DM [51,65,99].

*6.3.1.2 Combination Treatment.* The need for combination or new therapies is emphasised by the fact that one in seven DM patients on statins will ultimately experience a CV event over 5 years [98]. Additional benefits include not just greater lowering of LDL but a reduction in other cholesterol components, namely ApoB, Lp(a) and TG [112]. The residual risk of further CV events remains high even with optimal LDL lowering, emphasising the significance of treating other lipid derangements seen in these individuals [4].

#### 6.3.2 Ezetimibe

Ezetimibe inhibits dietary cholesterol absorption and the reabsorption of biliary cholesterol into the small intestine by interacting with the Niemann-Pick C1-like protein 1 (NPC1L1)] at the intestinal brush border. Upregulation of the hepatic LDLR expression in response to the reduced hepatic cholesterol delivery leads to increased LDL plasma clearance. Ezetimibe remains a second-line therapy in DM for reducing LDL where it is used in combination with statins when the therapeutic target is not achieved with statins at the maximal tolerated dose, or in individuals who are intolerant of statins [51].

The IMProved Reduction of Outcomes: Vytorin Efficacy International Trial (IMPROVE-IT) trial randomised participants with acute coronary syndrome (ACS) to a combination of simvastatin and ezetimibe or placebo. There was significant mean LDL reduction and overall absolute risk reduction in CV events in the combination treatment group [113]. This trial's findings suggest that LDL <2.0 mmol/L is the therapeutic goal in high-risk subjects even though the linear association between the proportional reduction in both CV risk and LDL implies that a lower limit for LDL does not exist [113], similar to the CTT trial findings [114]. Reducing LDL levels below former approved targets gave additional benefit, especially in patients ≥75 years and with DM [113].

The Simvastatin Heart and Renal Protection (SHARP) trial showed that LDL decrease (by 0.85 mmo/L) with simvastatin and ezetimibe reduced major ASCVD (by 17%) in advanced chronic kidney disease (CKD) patients safely and there was no significant difference between diabetics and non-diabetics [115].

#### 6.3.3 Bile acid sequestrant

Hepatic bile acids synthesised from cholesterol are released into the intestinal lumen where bile acid sequestrants bind to and prevent reabsorption of bile acids from the terminal ileum to be returned to the liver. Liver depletion of bile leads to increased bile acid synthesis from hepatic cholesterol leading to increased need for cholesterol in the liver. This, in turn, increases hepatic LDLR expression resulting in decreased plasma LDL [51]. Colesevelam lowers LDL, TC and non-HDL in addition to HbA1c [116]. Cholestyramine is better in LDL reduction. However, both have adverse gastrointestinal side effects, and raise TG, hence contraindicated if TG levels are >4.5 mmol/L as the risk of pancreatitis is increased [52].

In a 24-week randomised clinical trial, GOAL-RCT, among subjects with T2DM (HbA1c 7.1–10%, LDL > 2.0 mmol/L), the commencement of colesevelam or ezetimibe led to comparable within target results of LDL and HbA1c. However, ezetimibe achieved a greater reduction of LDL and was better tolerated than colesevelam [117].

#### 6.3.4 Nicotinic acid

Niacin lowers IDL, VLDL and LDL particles by inhibiting hepatic diacylglycerol acyl transferase-2 as well as increases HDL by stimulating hepatic ApoA1 production [51]. However, its side effects include hyperglycaemia and flushing [4].

The Atherothrombosis Intervention in MetS with Low HDL/High TG: Impact on Global Health Outcomes (AIM-HIGH) trial was terminated early as there was no significant difference in CVD outcomes from the addition of niacin to statin therapy [118]. In the Heart Protection Study 2-Treatment of HDL to Reduce the Incidence of Vascular Events (HPS2-THRIVE), extended-release niacin–laropiprant added to baseline lipid-lowering treatment among participants with ASCVD did not significantly decrease risk of CV events but there was an increase in adverse events [119]. In a meta-analysis of 11 randomised CT with 26,340 non-diabetics, niacin treatment was associated with a relative risk (RR) of 1.34 (95% CI 1.21–1.49) for new-onset diabetes, regardless of whether subjects were on background statin treatment or combination treatment with laropiprant [120]. As such, niacin-statin combination therapy is not recommended in DM as the harm outweighs its benefits [22].

#### 6.3.5 Fibrates

Fibrates activate the nuclear receptor peroxisome proliferator-activated receptor alpha (PPARα), abundant in the intestine, to reduce TG by increasing LPL activity and reducing ApoCIII [54]. PPARα, stimulated by polyunsaturated fatty acids, regulates SREBP-1c expression in fatty acid metabolism [6].

A pooled meta-analysis of 10 randomised CTs of 36,489 patients showed that long-term fibrate treatment significantly reduced non-fatal MI occurrence but had no significant consequence on other adverse CV outcomes [121]. Another meta-analysis of 18 trials on 45,058 participants concluded that fibrate treatment can lower major CV event (MACE) risk, primarily by coronary event prevention, and may have a role in patients with combined dyslipidaemia and increased CV risk [122].

Fibrates may have a role as adjunct therapy in T2DM with persistently elevated TG. When fasting TG is > 10 mmol/L, fibrate is advocated to lower the risk of pancreatitis [123]. Even though TG is not a therapeutic target for reduction in CV risk, an optimal TG concentration <1.5 mmol/L is associated with fewer lipid derangements, such as reduced HDL, sdLDL and postprandial lipaemia [65,68]. However, fenofibrate may cause a paradoxical reduction in HDL [52] and gemfibrozil in combination with statin is contraindicated as it predisposes to an increased risk of myopathy due to its inhibitory effect on statin metabolism via the glucuronidation pathway, leading to increased plasma statin concentration. Fibrates, being renally metabolised, should be avoided or used cautiously in CKD patients [124]. Fibrate treatment in ACCORD Lipid trial, similar to in FIELD showed a 13% reversible rise in creatinine with no detrimental effect on the kidneys and a positive outcome in diabetic retinopathy [125,126].

A phase III trial in T2DM patients demonstrated significant reduction in fasting TG and ApoB48 by 45% and 56%, respectively, for 52 weeks in the group treated with pemafibrate, a more potent and selective PPARα agonist with minimal adverse effects [127]. Combination fibrate-statin treatment in T2DM patients to reduce CV risk is discouraged by the FDA following the findings from the ACCORD Lipid trial [125]. However, a new large clinical trial, Pemafibrate to Reduce cardiovascular OutcoMes by reducing triglycerides IN diabetic patiENTs (PROMINENT) is currently ongoing in T2DM subjects with high TG, low HDL and suboptimal LDL reduction by statins to assess CV events [128,129].

#### 6.3.6 Omega-3 fatty acids

Omega-3 fatty acid formulations that contain docosahexaenoic acid (DHA) and eicosapentaenoic acid (EPA) [22] decrease TG in a dose-dependent fashion by acting on the GP120 receptor associated with PPARγ and anti-inflammation pathways with no effect on HDL and LDL [4].

In the Japan EPA Lipid Intervention Study (JELIS) of hypercholesterolaemic individuals, there was a significant reduction in CAD in the impaired glucose metabolism group as a result of EPA treatment [130]. The Reduction of Cardiovascular Events with Icosapent Ethyl–Intervention Trial (REDUCE-IT) trial concluded that in subjects with raised TG on statins, the risk of CV events and death was significantly reduced with EPA treatment [131].

Although omega-3 fatty acids have been shown to yield favourable effects on lipoprotein metabolism and thrombotic, inflammatory, vascular, arrhythmogenic and oxidative factors linked to CVD, the Outcome Reduction with an Initial Glargine (ORIGIN) trial demonstrated no effect of omega-3 fatty acids on CAD risk in subjects with IFG, IGT and T2DM [132]. The recent Long-Term Outcomes Study to Assess Statin Residual Risk with Epanova in High Cardiovascular Risk Patients with Hypertriglyceridemia (STRENGTH) trial findings also did not support the addition of Epanova, a carboxylic acid formulation of omega-3 fatty acids to background statin treatment to decrease CV events in high-risk individuals. The study was stopped prematurely when clinical benefit was evidently low and there was an increase in new-onset atrial fibrillation in the Epanova treatment group. However, whether benefits might be noted in a lower-risk primary prevention population remains to be elucidated [133].

#### 6.3.7 Proprotein convertase subtilisin/kexin 9 (PCSK9) inhibitors

Increased PCSK9 concentration decreases LDLR expression when PCSK9 binds to and causes lysosomal catabolism of LDLR leading to increased plasma LDL levels. Hence, using monoclonal antibodies, PCSK9 inhibitors reduce the levels of plasma PCSK9 that is then unavailable to bind to LDLR leading to decreased intracellular degradation and increased cell surface LDLR expression and, therefore, reduced plasma LDL levels [134].

Two approved PCSK9 inhibitors using human monoclonal antibodies, alirocumab and evolocumab, are given as subcutaneous injections every two to four weeks and significantly reduce LDL when given with a statin or alone [51]. Indications for use include ASCVD on maximal tolerated statins, in combination with ezetimibe or not, with LDL ≥1.8 mmol/L or non-HDL ≥ 2.6 mmol/L [135]. There are no adverse effects except that some patients can develop an injection site reaction. However, PCSK9 inhibitors are very expensive and can be a substantial burden on the economy [51,65,99,[101], [102], [103]].

TG transport and the metabolism of ApoB48, ApoB100, ApoCIII, and ApoE after a fat-rich meal were investigated before and on evolocumab treatment in 13 T2DM subjects. The study concluded that evolocumab had negligible effects on chylomicrons and VLDL1 (TG-carrying lipoproteins) but had a significant effect on VLDL2, IDL, LDL (cholesterol-carrying lipoproteins). PCSK9 inhibitors have little impact on TG transport in either the post-prandial or the fasting state [136].

A meta-analysis of 38 randomised CTs showed that PCSK9 inhibitors do not affect glucose metabolism and their effect on LDL and MACE in diabetic patients is comparable to that in non-diabetics. Treatment with PCSK9 inhibitors in diabetic patients is cost-effective considering the incidence of MACE in diabetics is approximately double that of the general population. Hence, this data suggests that diabetics should be treated more aggressively with PCSK9 inhibitors to achieve and maintain LDL lower than target values of non-diabetics [137].

A review on PCSK9 inhibitors for the primary and secondary CVD prevention included 24 randomised CTs on 60,997 participants (18 alirocumab and 6 evolocumab trials). Both drugs reduced CVD risk when added to other LDL-lowering treatment [138].

#### 6.3.8 MTP inhibitor

MTP transports phospholipids and TG from the endoplasmic reticulum to ApoB to form VLDL. Hence, MTP inhibition blocks hepatic VLDL and intestinal CM formation. The effect of lomitapide on CV outcomes has not been ascertained although it is used in treating homozygous familial hypercholesterolaemia [51].

#### 6.3.9 Antisense oligonucleotides to ApoB

After a subcutaneous injection, the antisense oligonucleotide, mipomersen is selectively transported to the liver where it binds to and triggers the degradation of a specific mRNA preventing ApoB protein translation. This, in turn, reduces production of atherogenic lipoproteins and lipids. Mipomersen is used in homozygous familial hypercholesterolaemia to lower LDL. The safety and efficacy of long-term mipomersen therapy are presently being evaluated in statin intolerant individuals [51].

### 6.4 Future drug developments

A future therapeutic option is dual agonists of both PPARα and PPARγ. Saroglitazar is the first approved dual PPAR agonist that reduces plasma TG, TC, VLDL, non-HDL, fasting glucose and HbA1c [139]. Gene therapy with alipogene tiparvovec has now been approved for LPL deficiency associated with T2DM caused by chronic pancreatitis [140].

Inclisiran, an investigational PCSK9-specific RNA silencing molecule with potential for a maintenance regimen of twice-yearly dosing, significantly lowered LDL and PCSK9 in the dose-ranging Trial to Evaluate the Effect of ALN-PCSSC Treatment on Low Density Lipoprotein Cholesterol (ORION-1 trial). In ORION-1, inclisiran treatment was added to subjects with ASCVD or its risk equivalents, and high LDL on maximally tolerated LDL lowering treatment. Inclisiran significantly lowered LDL and PCSK9 similarly in persons with and without diabetes at day 180. Temporal HbA1c were similar in the placebo and inclisiran arms. The inclisiran-treated groups also had lower ApoB, non-HDL, and Lp(a) but higher HDL. PCSK9-targeted small interfering (siRNA)-treatment may offer a novel treatment for dyslipidaemic subjects with or without DM [141].

Bempedoic acid inhibits ATP citrate lyase, a key enzyme upstream of HMG-CoA reductase, hence inhibiting cholesterol biosynthesis [51]. It has been assessed in diabetics [142], and statin intolerant subjects [143]. Bempedoic acid decreases LDL by approximately 30% when used as monotherapy and 50% in combination with ezetimibe [51]. Results of the Evaluation of the Efficacy and Safety of Bempedoic Acid as Add-on to Ezetimibe Therapy in Patients with Elevated LDL (CLEAR-Tranquility) trial revealed that bempedoic acid has acceptable safety and positive effects on lipid profile and hsCRP. Additional studies are required to determine its safety in the long-term [144].

Novel targets to reduce TG and cholesterol in diabetic dyslipidaemia include ApoCIII. ApoCIII inhibits LPL, thus increasing hepatic VLDL [6]. Volanesorsen, an antisense oligonucleotide therapy targeting ApoCIII mRNA has been developed and decreased plasma TG by 70% and ApoCIII by 80–90%. It has been approved for use in familial chylomicronaemia syndrome at high-risk for pancreatitis [145].

Various ANGPTLs such as ANGPTL3 and ANGPTL4 inhibit LPL activity causing an increase in plasma TG [146]. An ANGPTL3 antibody, Evinacumab under development reduces TG, Lp(a) and LDL in homozygous familial hypercholesterolaemia [147]. Inhibition of ANGPTL3 production by antisense oligonucleotide, IONIS-ANGPTL3-LRx is also being developed and decreases TG by 85% [148]. In fasting, ANGPTL4 expression is increased in adipose tissue inhibiting LPL activity, and hence, TG is redirected to other tissues for oxidation. Inactivating ANGPTL4 variants is associated with decreased CAD risk. ANGPTL4 is found in the intestine, and hence, may be a potential therapeutic target in regulation of CM secretion. Thus, novel therapeutic approaches may increase TRL clearance, which will be mirrored in the atherogenic load of remnant particles [6]. Table 2 summarises the significant findings from various clinical trials and meta-analysis on glucose-lowering and lipid-lowering treatments on prevention of CVD events.

**Table 2:** Clinical Trials and Meta-analysis demonstrating significant Primary and Secondary Prevention of CVD Events.

Drug Class	Clinical Trials	Participants	Intervention (versus placebo)	Main Outcomes*
PPARγ agonist	PRO-ACTIVE	5238 T2DM	Pioglitazone titrated 15 mg–45 mg od (+baseline medications)	Pioglitazone group vs. placebo: • had at least one event in 1° composite endpoint: HR 0.90, 95% CI 0.80–1.02. • reached 2° endpoint (composite of all-cause mortality, non-fatal MI, and stroke): HR 0.84, 0.72–0.98 [91].
SGLT2 inhibitors	EMPA-REG	7020 T2DM	Empagliflozin 10 mg or 25 mg od + standard therapy	Empagliflozin group vs. placebo: • occurrence of 1° outcome: HR 0.86, 95% CI 0.74–0.99 • occurrence of 2° outcome: HR 0.89, 95% CI, 0.78–1.01 • death from CV causes: HR 0.62, 95% CI 0.49–0.77; 38% RR reduction. • hospitalisation for heart failure: 35% RR reduction. • death from any cause: 32% RR reduction [93].
GIT lipase inhibitor	XENDOS	3305 non-DM BMI >30 kg/m^2^	Orlistat 120 mg tid + lifestyle changes	Orlistat + lifestyle changes vs. placebo: • Progression to T2DM: HR 0.627, 95% CI 0.455–0.863; 37.3% RR reduction [95].
Statins	CTT Met-analysis	18,686 (1466 T1DM 17,220 T2DM)	14 randomised CT	Statins vs. placebo ((RR per mmol/L LDL reduction): In T2DM: • all-cause mortality: RR 0.91, 99% CI 0.82–1.01 • reduction in CHD mortality: RR 0.88, 99% CI 0.75–1.03 • reduction in vascular mortality: RR 0.87, 99% CI 0.76-1.00 • incidence of major vascular event: RR 0.79, 99% CI 0.72–0.86 • incidence of major coronary event: RR 0.78, 99% CI 0.69–0.87 • incidence of coronary revascularisation: RR 0.75, 99% CI 0.64–0.88 • incidence of stroke: RR 0.79, 99% CI 0.67–0.93 In T1DM: • reduction in major vascular events: RR 0.79, 99% CI 0.62–1.01 [98].
Ezetimibe	IMPROVE-IT	18,144 (27% T2DM)	Simvastatin 40 mg + Ezetimibe 10 mg od	Simvastatin–Ezetimibe group vs. Simvastatin group: • occurrence of 1° end point (composite of CV death, non-fatal MI, unstable angina requiring rehospitalisation, coronary revascularisation or non-fatal stroke): HR 0.936; 95% CI 0.89–0.99 [113].
Fibrates	Meta-analysis	36,489	10 randomised CT (2 trials in T2DM only)	Fibrate group vs. placebo: • occurrence of non-fatal MI: OR 0.78, CI 0.78–0.86 • ↓TC 8% and ↓ TG by 30% • ↑ HDL by 9% [121].
	Meta-analysis	45,058	18 randomised CT (6 trials in T2DM only)	Fibrate group vs. placebo: • MACE: 10% RR reduction, 95% CI 0-18 • coronary events: 13% RR reduction, 95% CI 7-19 • risk of albuminuria progression: 14% RR reduction, 95% CI 2–25 [122].
Omega-3 fatty acid	JELIS	18,645 (16% DM)	Pravastatin 10 mg or Simvastatin 5 mg od + EPA 600 mg tid	Impaired glucose metabolism group had a significantly higher CAD HR compared to normoglycaemic group: • non-EPA group: HR 1.71, 95% CI 1.37–2.13 • EPA group: HR 1.63, 95% CI 1.27–2.09 [130].
	REDUCE-IT	8179 (59% DM)	Baseline statin + EPA 4 g od	Icosapent ethyl group vs. placebo: • occurrence of 1° end point: HR 0.75, 95% CI 0.68–0.83 • occurrence of 2° end point: HR 0.74, 95% CI 0.65–0.83 [131].
PCSK9 inhibitors	Meta-analysis	46,833 (PCSK9 inhibitors) & 42,770 (comparator) (21.5% T2DM)	38 randomised CT on PCSK9 inhibitors vs placebo/any comparator	PCSK-9 inhibitors vs. placebo or any comparator: • incidence of MACE: - Diabetics: OR 0.82, 95% CI 0.74–0.91 - Non-diabetics: OR 0.73, 95% CI 0.64–0.84 [137].
	Review of studies	60,997	24 randomised CT on Alirocumab (18) & Evolocumab (6)	Alirocumab vs. placebo (high-certainty evidence) - decreased the risk of: • CVD events: ARD -2%; OR 0.87, 95% CI 0.80–0.94 • mortality: ARD -1%; OR 0.83, 95% CI 0.72–0.96 • MI: ARD -2%; OR 0.86, 95% CI 0.79–0.94; • any stroke: ARD 0%; OR 0.73, 95% CI 0.58–0 Alirocumab vs. ezetimibe & statins (low-certainty evidence) - decreased the risk of: • CVD events: ARD 1%; OR 1.37, 95% CI 0.65–2.87 • mortality: ARD -1%; OR 0.51, 95% CI 0.18–1.40 • MI: ARD 1%; OR 1.45, 95% CI 0.64–3.28 • any stroke: ARD < 1%; OR 0.85, 95% CI 0.13–5.61 Evolocumab vs. placebo (high-certainty evidence) - decreased the risk of: • CVD events: ARD -2%; OR 0.84, 95% CI 0.78–0.91 • mortality: ARD < 1%; OR 1.04, 95% CI 0.91–1.19 • MI: ARD -1%; OR 0.72, 95% CI 0.64–0.82 • any stroke: ARD < −1%; OR 0.79, 95% CI 0.65–0.94 Evolocumab vs. ezetimibe & statins (low-certainty evidence) - decreased the risk of: • CVD events: ARD < −1%; OR 0.66, 95% CI 0.14–3.04 • mortality: ARD < 1%; OR 0.43, 95% CI 0.14–1.30 • MI: ARD < 1%; OR 0.66, 95% CI 0.23–1.85 • any stroke: insufficient data [138].

*all results have significant p-value.

**Abbreviations:** ARD, absolute risk difference; BMI, body mass index; CV, cardiovascular; CVD, cardiovascular disease; CT, clinical trial; CI, confidence interval; CAD, coronary artery disease; ↓, decrease; DM, diabetes mellitus; EPA, eicosapentaenoic acid; GIT, gastrointestinal; HR, hazard ratio; HDL, high density lipoprotein cholesterol; ↑, increase; LDL, low density lipoprotein cholesterol; od, daily; MACE, major cardiovascular event; MI, myocardial infarction; OR, odds ratio; PPARγ, peroxisome proliferator-activated receptor gamma; 1°, primary; PCSK9, proprotein convertase subtilisin/kexin 9; RR, relative risk; 2°, secondary; SGLT2, sodium-glucose cotransporter 2; tid, three times daily; TC, total cholesterol; TG, triglyceride; T1DM, type 1 diabetes mellitus; T2DM, type 2 diabetes mellitus; vs, versus.

## 7 Guidelines

Various international guidelines have been developed on lipid management in DM, focusing on CV risk reduction such as the 2020 American Association of Clinical Endocrinologists and American College of Endocrinology (AACE/ACE) on the Management of Dyslipidemia and Prevention of Cardiovascular Disease Algorithm – 2020 Executive Summary [99], the 2019 European Society of Cardiology/European Atherosclerosis Society (ESC/EAS) Guidelines for the management of dyslipidaemias: lipid modification to reduce cardiovascular risk: The Task Force for the management of dyslipidaemias of the European Society of Cardiology (ESC) and European Atherosclerosis Society (EAS) [51], 2019 American Diabetes Association (ADA): Cardiovascular Disease and Risk Management: Standards of Medical Care in Diabetes – 2019 [103], 2018 American Heart Association/American College of Cardiology/Multisociety: 2018 AHA/ACC/AACVPR/AAPA/ABC/ACPM/ADA/AGS/APhA/ASPC/NLA/PCNA Guideline on the Management of Blood Cholesterol: A Report of the American College of Cardiology/American Heart Association Task Force on Clinical Practice Guidelines [102], Canadian Diabetes Association (CDA): 2018 Diabetes Canada Clinical Practice Guidelines Expert Committee on Dyslipidaemia [65], 2017 Australian Diabetes Society (ADS) on the 2017 Australian Guidelines for use of Lipid Lowering Therapy in Diabetes [101] and National Institute for Health and Care Excellence (NICE) 2014 clinical guideline on Cardiovascular disease: risk assessment and reduction, including lipid modification [104]. However, they have different approaches in achieving the common underlying objective in terms of procedures of risk assessment, classification of risk cohorts, and thresholds for therapy and corresponding therapeutic goals (Table 3). NICE guideline is the only guideline that considers socioeconomic status as an additional factor that contributes to CVD risk [104].

**Table 3:** International guidelines on management of dyslipidaemia in diabetes mellitus.

2020 AACE/ACE [99]	2019 ESC/EAS [51]	2019 ADA [103]	2018 AHA/ACC/Multisociety [102]	2018 CDA [65]	2017 ADS [101]	2014 NICE [104]
*Extreme risk:* Established clinical ASCVD −high-intensity statin or highest tolerated statin dose) to achieve goals: LDL < 1.4 mmol/L non-HDL < 2.1 mmol/L ApoB < 0.7 g/L −If LDL > goal after 3 months, add 2^nd^ line treatment and a 3^rd^ drug should be added if there is failure to achieve the goal with combination treatment.	High-intensity statin up to highest tolerated dose to achieve goals specific to risk category. −If the goals not achieved, add combination therapy. −In T1DM who are at high or very-high risk, treat with statins.	*All diabetics with ASCVD or 10-year CV risk > 20%:* −high-intensity statins (≥ 50% decrease in LDL). −If LDL ≥ 1.8 mmol/L despite maximally tolerated statin dose, use combination treatment.	*20 -39 years: with independent DM-specific risk enhancers* −moderate intensity statin.	Statins should be used to reduce CV risk in T1DM or T2DM with any of the following features: 1) clinical CVD 2) age ≥ 40 years 3) Age < 40 years and 1 of the following: i) diabetes duration > 15 years and age > 30 years ii) microvascular complications iii) warrants treatment based on presence of other CV risk factors.	When using absolute risk to guide treatment, CV risk in the next 5 years defined as low (< 10%), moderate (10-15%) or high (> 15%).	1° CVD prevention, start atorvastatin 20 mg to achieve non-HDL reduction > 40% in: •T1DM who: - > 40 years or - diabetes > 10 years or - established nephropathy or - other CVD risk factors. •T2DM who have *10-year CV risk ≥ 10%.*

*Very high risk:* with ≥ 1 risk factor(s) −add 2^nd^ line therapy to achieve LDL goal for patients who require < 15-20% further decrease to achieve goals: LDL < 1.8 mmol/L non-HDL < 2.6 mmol/L ApoB < 0.8 g/L	*Very high risk:* target organ damage, or at least 3 major risk factors, or early onset T1DM > 20 years. −LDL reduction ≥ 50% from baseline and goals: LDL < 1.4 mmol/L non-HDL < 2.2 mmol/L ApoB < 0.65 g/L	*Age < 40 with additional CV risk factors, age 40-75 years and > 75 years without ASCVD or 10-year CV risk* *< 20%:* −moderate intensity statins (30%-49% decrease in LDL).	*40 -75 years: irrespective of 10-year CV risk* −moderate-intensity statin.	−statin to achieve LDL < 2.0 mmol/L or LDL reduction ≥ 50% from baseline. Other goals: ApoB < 0.8 g/L non-HDL < 2.6 mmol/L −If not achieved, use combination therapy.	*Existing CVD or high risk:* −high intensity statin (LDL reduction ≥ 50% from baseline and LDL < 1.8 mmol/L).	If non-HDL < 40% reduction after 3 months: - consider increasing dose if started on < 80 mg atorvastatin.

*High risk:* with no other risk factors −moderate-intensity statin then increase to high-intensity statin before adding a 2^nd^ line treatment to achieve goals: LDL < 2.6 mmol/L non-HDL < 3.4 mmol/L ApoB < 0.9 g/L	*Very high risk with recurrent ASCVD events*: −Goals: non-HDL < 1.8 mmol/L ApoB < 0.55 g/dL		*> 75 years:* −already on statins, continue therapy.		*Moderate risk:* −moderate intensity statin and LDL reduction ≥ 30% from baseline) and LDL < 2.0 mmol/L.	*Existing CVD:* atorvastatin 80 mg or highest tolerated dose.

TG < 1.7 mmol/L for all risk categories.	*High risk:* without target organ damage, DM duration ≥ 10 years or another risk factor. −LDL reduction ≥ 50% from baseline and goals: LDL < 1.8 mmol/L non-HDL < 2.6 mmol/L ApoB < 0.8 g/L		*Have multiple ASCVD risk factors*: −high-intensity statin (LDL reduction ≥ 50% from baseline).		Combination therapy if targets are not reached.	

	*Moderate risk:* T1DM < 35 years and T2DM < 50 years with DM duration < 10 years, without other risk factors. −Goals: LDL < 2.6 mmol/L non-HDL < 3.4 mmol/L ApoB < 1.0 g/L		*10-year CV risk* ≥ *20%*: combination therapy with maximally tolerated statin therapy (LDL reduction ≥ 50% from baseline).		Add fenofibrate when LDL at target but TG ≥ 2.3 mmol/L and HDL < 1.0 mmol/L (male); < 1.29 mmol/L (female) or in all T2DM patients with existing retinopathy.	

	TG < 1.7 mmol/L for all risk categories.					

**Abbreviations:** AACE/ACE, American Association of Clinical Endocrinologists and American College of Endocrinology; ADA, American Diabetes Association: Cardiovascular Disease and Risk Management: Standards of Medical Care in Diabetes; ADS, Australian Diabetes Society; AHA/ACC/Multisociety, American Heart Association/American College of Cardiology/Multisociety, ApoB, apolipoprotein B; ASCVD, atherosclerotic cardiovascular disease; CDA, Canadian Diabetes Association; CV, cardiovascular; CVD, cardiovascular disease; DM, diabetes mellitus; ESC/EAS, European Society of Cardiology/European Atherosclerosis Society; LDL, low density lipoprotein cholesterol; NICE, National Institute for Health and Care Excellence; non-HDL, non-high density lipoprotein cholesterol; 1°, primary; TG, triglyceride; T1DM, type 1 diabetes mellitus; T2DM, type 2 diabetes mellitus.

All guidelines strongly recommend lifestyle modification in addition to statin (moderate or high intensity) as the first-line treatment [51,65,99,[101], [102], [103], [104]]. The statin therapy should be at maximum intensity before the introduction of combination treatment. In statin intolerance or in those who do not achieve LDL goal while on maximally tolerated statin, ezetimibe or PCSK9-inhibitor can be added. Ezetimibe is preferred by all guidelines due to lower cost [51,65,99,[101], [102], [103]]. The ESC/EAS guidelines, however, offer more liberal suggestions for addition of PCSK9 inhibitors [51].

All guidelines [51,99,[101], [102], [103]] except CDA [65] and NICE [104] recommend risk stratification of subjects with further subdivision based on risk factors/risk enhancers. The main aim of all guidelines is to reduce ASCVD risk [51,65,99,[101], [102], [103], [104]] and LDL level is used as the primary therapeutic goal [51,65,99,[101], [102], [103]].

The intensity of lipid-lowering therapy depends on the absolute ASCVD risk. All guidelines recommend a reduction in LDL ≥50% from baseline with treatment [51,65,99,[101], [102], [103]] except the NICE guideline, which recommends non-HDL reduction ≥ 40% from baseline with treatment. In the NICE guideline, non-HDL is considered a better CVD risk indicator than LDL, being more accurate, practical and cost effective. Furthermore, a fasting blood sample is not required, which is more convenient for patients [104]. The ESC/EAS and AACE/ACE guidelines include achievement of both the LDL percentage reduction as well as absolute LDL target values specific for risk groups. For each risk category in the AACE/ACE and ESC/EAC guidelines, there are treatment goals to be achieved for LDL, non-HDL, and ApoB levels, proportional to the degree of risk. A goal for TG is also offered [51,99]. The CDA guidelines also provides therapeutic goals for non-HDL and ApoB [65].

The differences in various guidelines may confuse clinicians. The 2013 ACC/AHA guideline, however, states, “*Recommendations are designed to inform clinical judgment, not replace it*” [149]. Among all the guidelines, the lowest LDL target has been recommended by both the AACE/ACE and ESC/EAS guidelines, which is < 1.4 mmol/L in extreme risk and very-high risk T2DM, respectively [51,99]. This LDL lowering beyond the previous set goals was justified by ESC/EAC as being associated with fewer CV events [51]. By comparing these society guidelines, clinicians can become more aware of the context of these recommendations to help provide the best individualised treatment for their patients.

## 8 Conclusion

Much of the pathophysiology linking DM and dyslipidaemia has been elucidated. Although undoubtedly of importance, diabetic dyslipidaemia is likely to be only one of many reasons for the accelerated macrovascular disease in diabetic patients. Nonetheless, the lowering in LDL is strongly associated with decrease in CV risk. The significant decrease in CV events with statin therapy outweighs the slightly increased risk of developing T2DM. It is important to understand the underlying mechanisms of diabetic dyslipidaemia in order to develop new therapeutic strategies against dyslipidaemia and diabetes. The various international guidelines on lipid management can be used to tailor an appropriate approach to each patient with diabetes and dyslipidaemia.

## Funding

This review did not receive any specific grant from funding agencies in the public, commercial, or not-for-profit sectors.

## Authors’ contributions

**Subashini C. Thambiah:** Investigation, Writing – Original draft preparation, Reviewing and Editing. **Leslie Charles Lai:** Conceptualisation, Supervision, Writing – Reviewing and Editing. Both authors approved the final submission.

## Declaration of competing interest

The attached revised manuscript has been read and approved by both authors **and there is no conflict of interest.** This revised article has not been submitted to any other journal for publication.
